# Identification of the expression patterns and potential prognostic role of m6A-RNA methylation regulators in Wilms Tumor

**DOI:** 10.1186/s12920-023-01660-2

**Published:** 2023-09-21

**Authors:** Changlin Jia, Hongjie Gao, Wenyue Ma, Xiaoya Liu, Mengmeng Chang, Fengyin Sun

**Affiliations:** 1 Department of Pediatric Surgery, Qilu Hospital of Shandong University, Jinan, Shandong China; 2 Department of Pediatrics, Qilu Hospital of Shandong University, Jinan, Shandong China

**Keywords:** m6A-RNA methylation, Wilms Tumor, m6Ascore, Prognosis, Tumor immune microenvironment

## Abstract

**Background:**

To explore the potential role of m6A methylation modification in Wilms Tumor (WT) by m6A-RNA Methylation (m6A) regulators.

**Methodology:**

The association of m6A modification patterns with immune and prognostic characteristics of tumors was systematically evaluated using 19 m6A regulators extracted from Wilms Tumor’s samples in public databases. A comprehensive model of “m6Ascore” was constructed using principal component analysis, and its prognostic value was evaluated.

**Results:**

Almost all m6A regulators were differentially expressed between WT and normal tissues. Unsupervised clustering identified three distinct m6A clusters that differed in both immune cell infiltration and biological pathways. The m6Ascore was constructed to quantify m6A modifications in individual patients. Our analysis suggests that m6Ascore is an independent prognostic factor for WT and can be used as a novel predictor of WT prognosis.

**Conclusions:**

This study comprehensively explored and systematically characterized m6A modifications in WT. m6A modification patterns play a critical role in the tumor immune microenvironment (TIME) and WT prognosis. m6Ascore provides a more comprehensive understanding of m6A modifications in WT and offers a practical tool for predicting WT prognosis. This study will help clinicians to identify valid indicators of WT to improve the poor prognosis of this disease.

**Supplementary Information:**

The online version contains supplementary material available at https://www.aliyundrive.com/drive/folder/64be739cd6956a741fb24670baeea53422be6024.

**Supplementary Information:**

The online version contains supplementary material available at 10.1186/s12920-023-01660-2.

## Introduction

Renal tumors are the fifth most common tumor in children, and Wilms Tumor (WT) is the most common renal malignancy in children [1]. The long-term survival rate of WT has steadily increased over the past decades to 85% and has even reached 90% in developed countries [2–4]. However, nearly 15% of patients still experience recurrence and associated complications [5–7]. Genes such as *WT1*, *WT2* and *MYCN* family have been suggested to be involved in the development of WT, and the detection of *CTR9*, *DICER1*, *REST, TP53*, *TRIM28* and *WT1* have been recommended as effective predictors of WT [3]. In addition, *SPRY1*, *SPIN4*, *MAP7D3*, *C10orf71*, and *SPAG* have also been analyzed for WT [8]. Recent studies suggest that cholesterol markers and methylation modifications may potentially influence WT [9, 10]. However, none of these studies could be included in the individualized assessment of specific patients. Newly discovered markers in WT are increasing, and these may eventually play a role in targeted therapies; unfortunately, only one genetic biomarker, *LOH* at chromosome 1p/16q, has been used in clinical therapy [11]. Thus, there is an urgent need to find effective biomarkers to predict the prognosis of WT and to develop new targets for WT therapy.

RNA modifications are universal post-transcriptional modifications that play a crucial role in biological regulation [12, 13]. N6-methyladenosine (m6A) is the most abundant epigenetic-transcriptomic modification in eukaryotic mRNAs [14]. As a reversible RNA modification, m6A plays an important role in the regulation of biological processes such as RNA degradation and splicing [15]. There is increasing evidence that m6A modifications play an important role in tumorigenesis and tumor regulation. *METTL14*, a regulator of m6A, has been reported to affect WT progression and prognosis by regulating related gene expression and splicing patterns [16], and three different multicenter case-control studies have suggested that m6A regulators *ALKBH5*, *YTHDF1* and *YTHDF2* all affect WT progression and prognosis to some extent [17–19]. Recently, m6A alterations have been shown to be present and to influence tumorigenesis and prognosis in a variety of tumors, including colon, lung, pancreatic, cervical, ovarian, nasopharyngeal, and prostate cancers [20–26].

In the present study, we systematically evaluated m6A modification patterns and the tumor immune microenvironment (TIME) in WT patients. We identified three distinct m6A modification patterns in WT; these clusters differed significantly in prognosis, immune cell infiltration and biological pathways. Based on m6A regulators and related genes, we constructed a model (called “m6Ascore”) to quantify the m6A modification patterns in individual patients. This study also suggests that m6Ascore may be a new practical tool to predict the prognosis of WT.

## Materials and methods

### Data acquisition and processing

The RNA sequencing data of Wilms Tumor (2018) were obtained from the Cancer Genome Atlas database (TCGA, https://portal.gdc.cancer.gov/). Gene expression data (measured in fragments per kilobase of exon per million fragments mapped or FPKM) were converted to transcripts per kilobase million (TPM).

### Analysis of m6A regulators in Wilms Tumor

Based on previous studies, we obtained 19 m6A regulators [27]. These regulators included 7 “Writer” (*METTL3*, *METTL14*, *WTAP*, *VIRMA*, *METTL16*, *RBM15*, and *RBM15B*), 9 “Reader” (*YTHDF1*, *YTHDF2*, *YTHDF3*,*YTHDC1*,*YTHDC2*,*IGF2BP1*,*IGF2BP2*,*IGF2BP3* and *HNRNPA2B1*), and 3 “Eraser” (*FTO*,*ALKBH5* and *ALKBH1*). The expression profiles of these regulators were systematically extracted and analyzed in normal and tumor samples. Somatic mutations of WT were assessed using the R package “maftools”. Tumor mutational burden (TMB) was calculated and the correlation of TMB with clinical features was evaluated. The prognostic value of m6A regulators was assessed using Kaplan-Meier (KM) curves and log-rank tests.

### Clustering analysis based on m6A regulators

Based on the expression of m6A regulators, unsupervised clustering was performed using the “ConsensusClusterPlus” R package root to identify different m6A modification patterns in WT patients, and the stability of the clusters was ensured by 1,000 replicates. Survival analysis of different clusters was performed using the KM method. Differences in biological processes between clusters were investigated by gene set variation analysis (GSVA) using the R package “GSVA”. The gene set “c2.cp.kegg. v7.4. symbol” was obtained from the Molecular Signatures Database (MSigDB). An adjusted p-value < 0.05 was considered statistically significant.

### Comparison of tumor immune microenvironment between different m6A clusters

Single sample gene set enrichment analysis (ssGSEA) was used to quantify the relative infiltration levels of 29 immune cell types in WT samples. The proportion of immune stromal components in the tumor microenvironment (TME) was measured using the " estimate” R package. Wilcoxon rank sum test was used to analyze differences in TME between clusters. In addition, the “limma” R package was used to examine differences in the expression of molecules such as targeted immune checkpoints between different functional clusters.

### Prognostic differential expression of genes between different m6A clusters

Principal component analysis (PCA) was used to investigate the different m6A modification patterns in WT. The empirical Bayesian approach was used to extract the differentially expressed genes (DEGs) between the different m6A clusters. The significance criterion for DEGs was set to adjusted p-value < 0.05. Gene Ontology (GO) biological process analysis and Kyoto Encyclopedia of Genes and Genomes (KEGG) pathway analysis were used to explore the rich functional annotation of DEGs [28]. Univariate Cox regression analysis was performed to examine the prognostic value of each DEG. Significance criteria were set at p-value < 0.05 and abs (logFC) > 1.

### Construction of m6Ascore

A PCA-based scoring system (called “m6Ascore”) was constructed to quantify the m6A modification pattern of individual WT patients. Principal components 1 and 2 and 3 were selected as signature scores. m6Ascore was defined using a method similar to the Genomic Grade Index (GGI) [29, 30]: m6Ascore=∑(PC1i + PC2i + PC3i), where i is the expression of DEG with prognostic efficacy in different m6A clusters. The samples were divided into high and low m6Ascore groups based on the scores. Correlation analysis was performed to investigate the relationship between m6Ascore and a number of relevant biological pathways, including immune correlation analysis, clinical correlation analysis, TMB and targeted immune checkpoint molecules. The prognostic value of m6Ascore was assessed using KM curves. Univariate and multivariate independent prognostic analyses were performed to evaluate whether the model was an independent prognostic factor for WT.

### Statistical analysis

All statistical analyses were performed with R software (version 4.1.2). Wilcox test was used to compare m6A regulators expression levels in WT tissues. Patient survival was dichotomized for continuous variables using optimal cutoff values determined by the R package “survminer”. For prognostic analysis, survival curves were constructed using the KM method, and log-rank tests were used to determine the significance of differences. Receiver operating characteristic (ROC) curves (R package “timeROC”) and area under the curve (AUC) values were used to assess the prognostic value of the m6Ascore. Univariate and multivariate independent prognostic analyses were performed to evaluate whether the model was an independent prognostic factor for WT. All statistical p-values were two-sided, and p < 0.05 was considered statistically significant.

## Results

### Differential expression of m6A regulators in Wilms Tumor

**Fig. 1:**
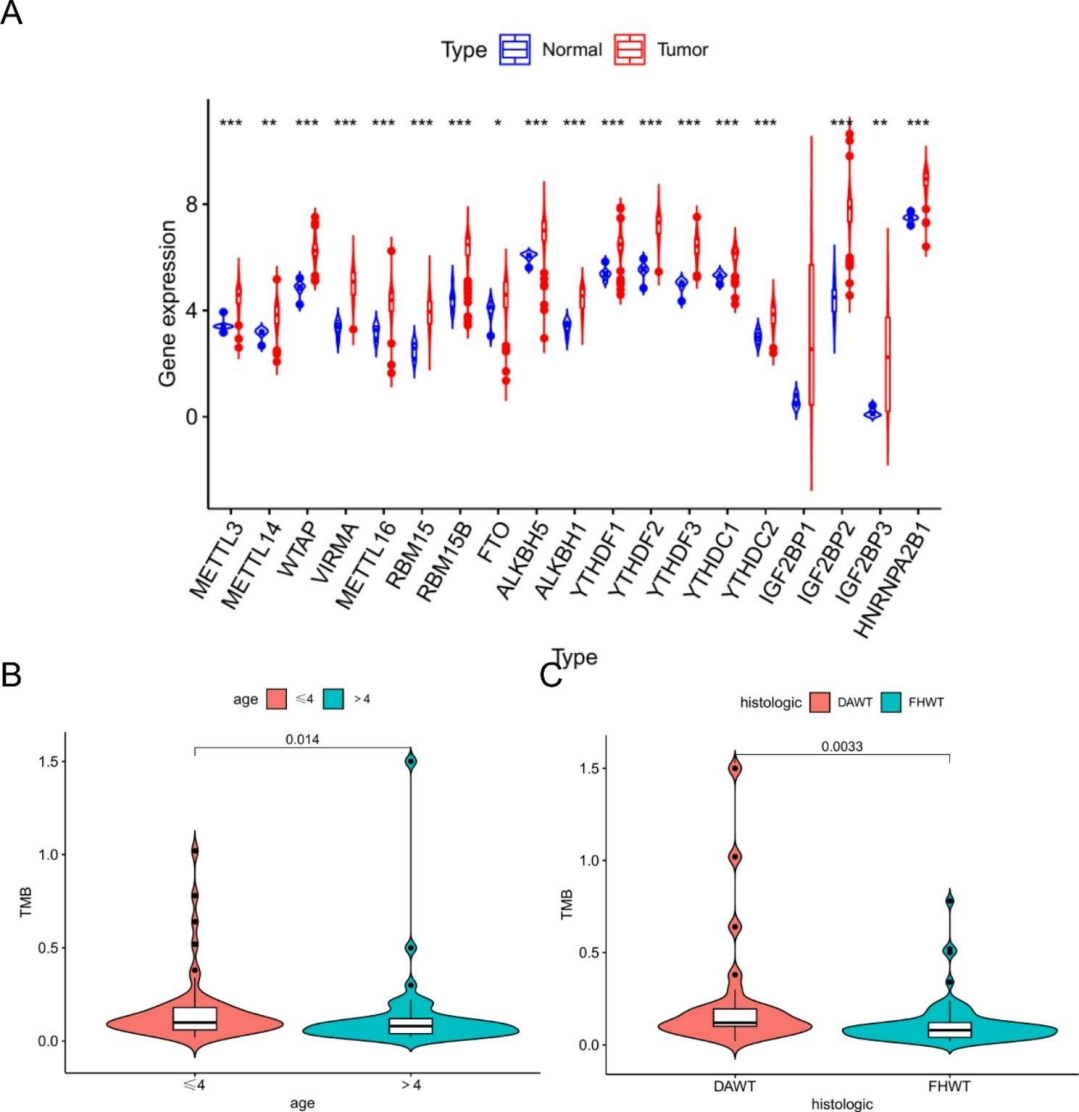
**A**: m6A modulator factor expression in tumor tissues vs. normal tissues; **B-C**: TMB vs. different clinical features

We obtained expression data from the TCGA database for a total of 132 samples, including 6 normal tissues and 126 tumor tissues. The results showed significant differences in the expression of almost all m6A regulators between tumor and normal tissues, with the majority of m6A regulators being upregulated in WT tissues (P < 0.001), while *ALKBH5* and *YTHDC1* were downregulated in WT expression levels (Fig. 1.A). In addition, our results showed that TMB in WT patients differed with different clinical characteristics, such as younger age group and diffusely anaplastic Wilms Tumor (DAWT) had higher TMB (Fig. 1.B-C).

**Fig. 2:**
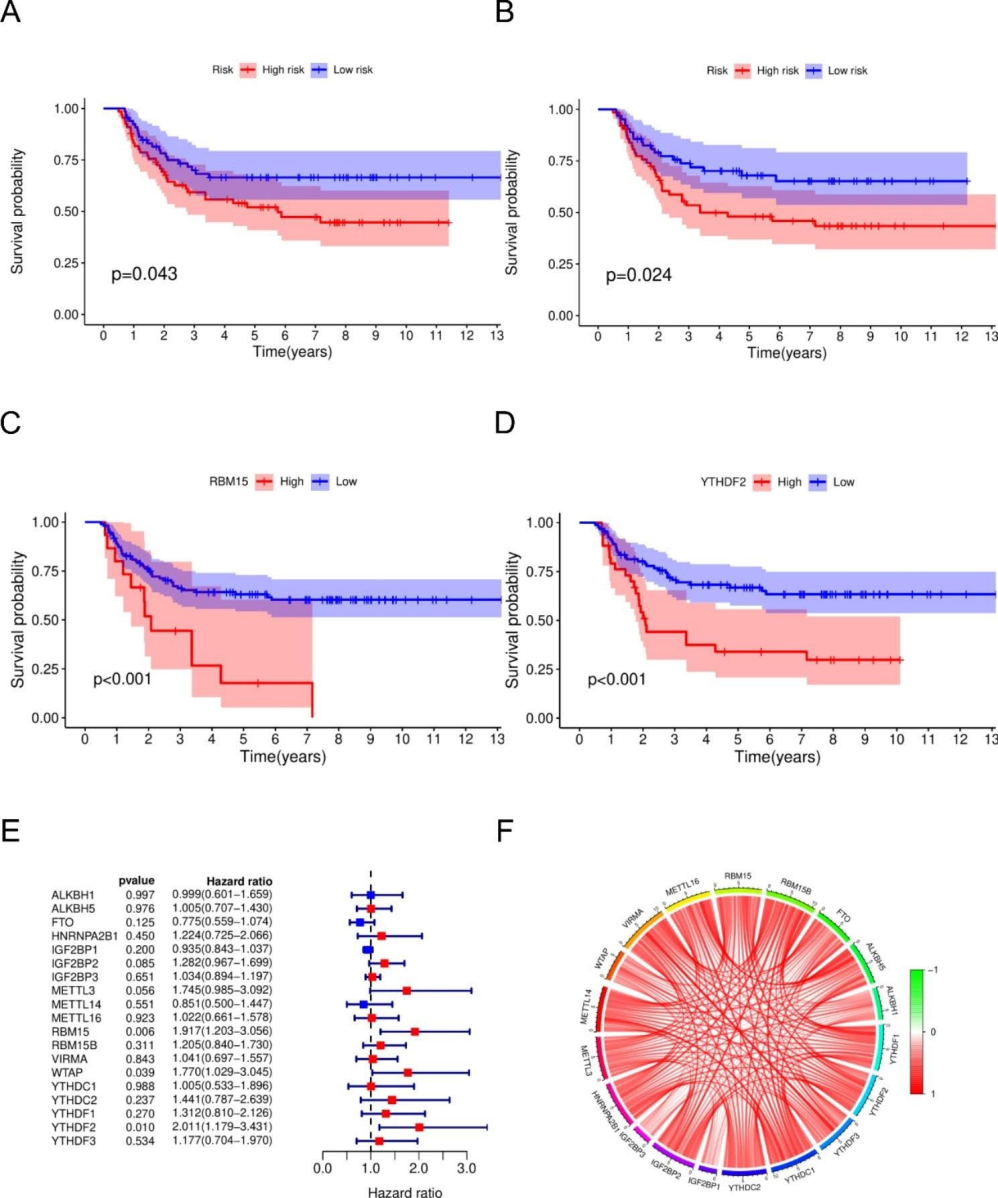
**A-D**:KM curves of m6A regulators; **E**: COX regression analysis of m6A regulators; **F**: Correlation of m6A regulator expression

Univariate COX regression analysis and KM showed that m6A modulators were potential prognostic factors for WT patients, such as *RBM15*, *WTAP* and *YTHDF2* showed high tumorigenicity (Fig. 2.A-E). There was also a significant positive correlation between each m6A modulator (Fig. 2.F). In conclusion, m6A regulators showed significant heterogeneity and differential expression in WT tissues compared with normal tissues, and m6A regulators may play a critical role in the development and progression of WT.

### m6A modification patterns based on m6A regulators

**Fig. 3:**
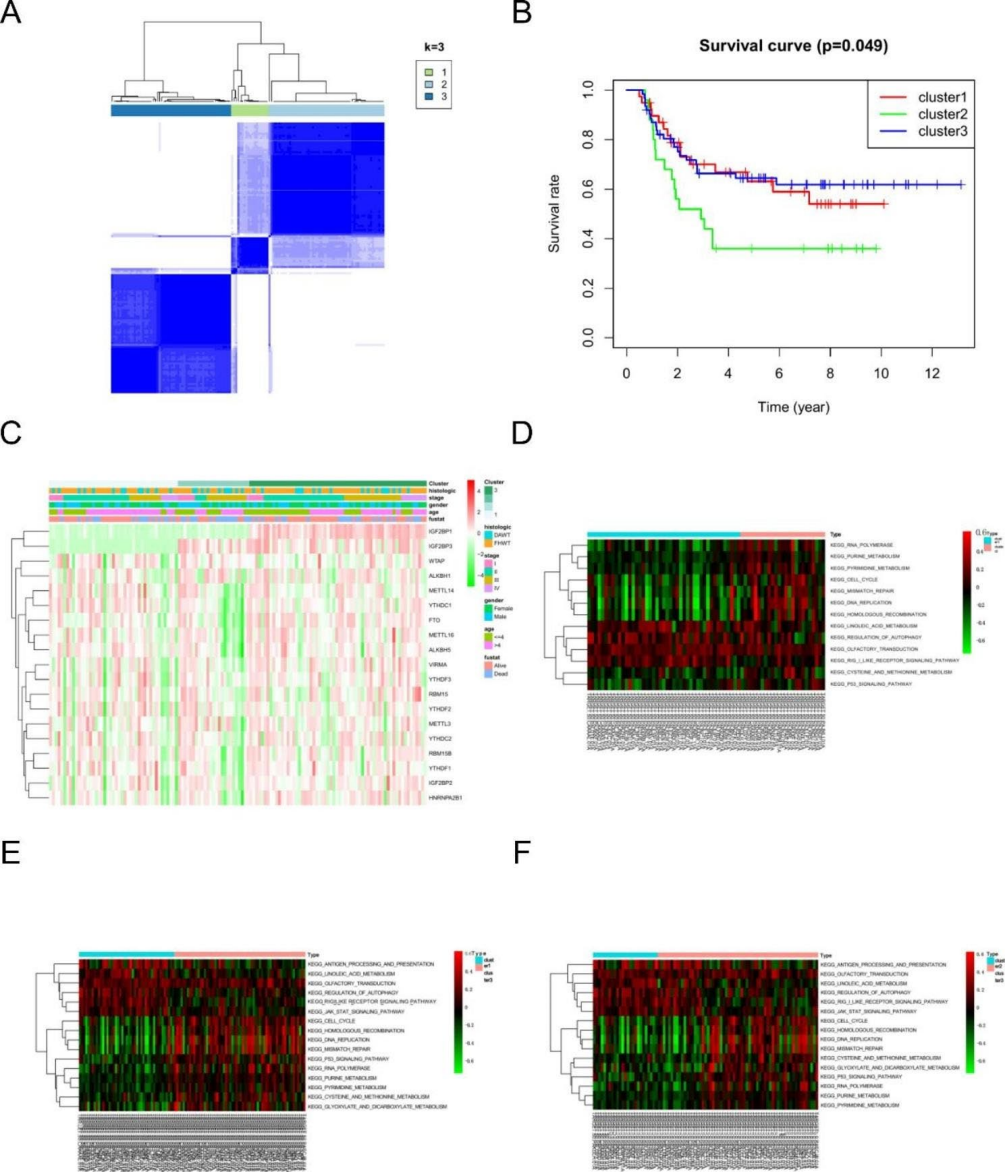
**A**: clustering analysis based on m6A regulators; **B**: survival analysis of different modification patterns; **C**: m6A regulator expression of different modification patterns; **D-F**: GSVA analysis of different modification patterns

Based on the expression of 19 m6A regulators, unsupervised cluster analysis was performed and three different m6A modification patterns (clusters 1–3) were identified, with 39 cases in cluster1, 25 cases in cluster2 and 62 cases in cluster3 (Fig. 3.A). Survival analysis showed some differences in prognosis between the different modification patterns (Fig. 3.B). Further analysis showed differences in regulator expression in the 3 different m6A alteration patterns (Fig. 3.C). In addition, we grouped the three different modification patterns into two vehicles for GSVA analysis, and the results showed significant differences in biological behavior between the different modification patterns (Fig. 3.D-F).

### Immunological characteristics of different m6A modification patterns

**Fig. 4:**
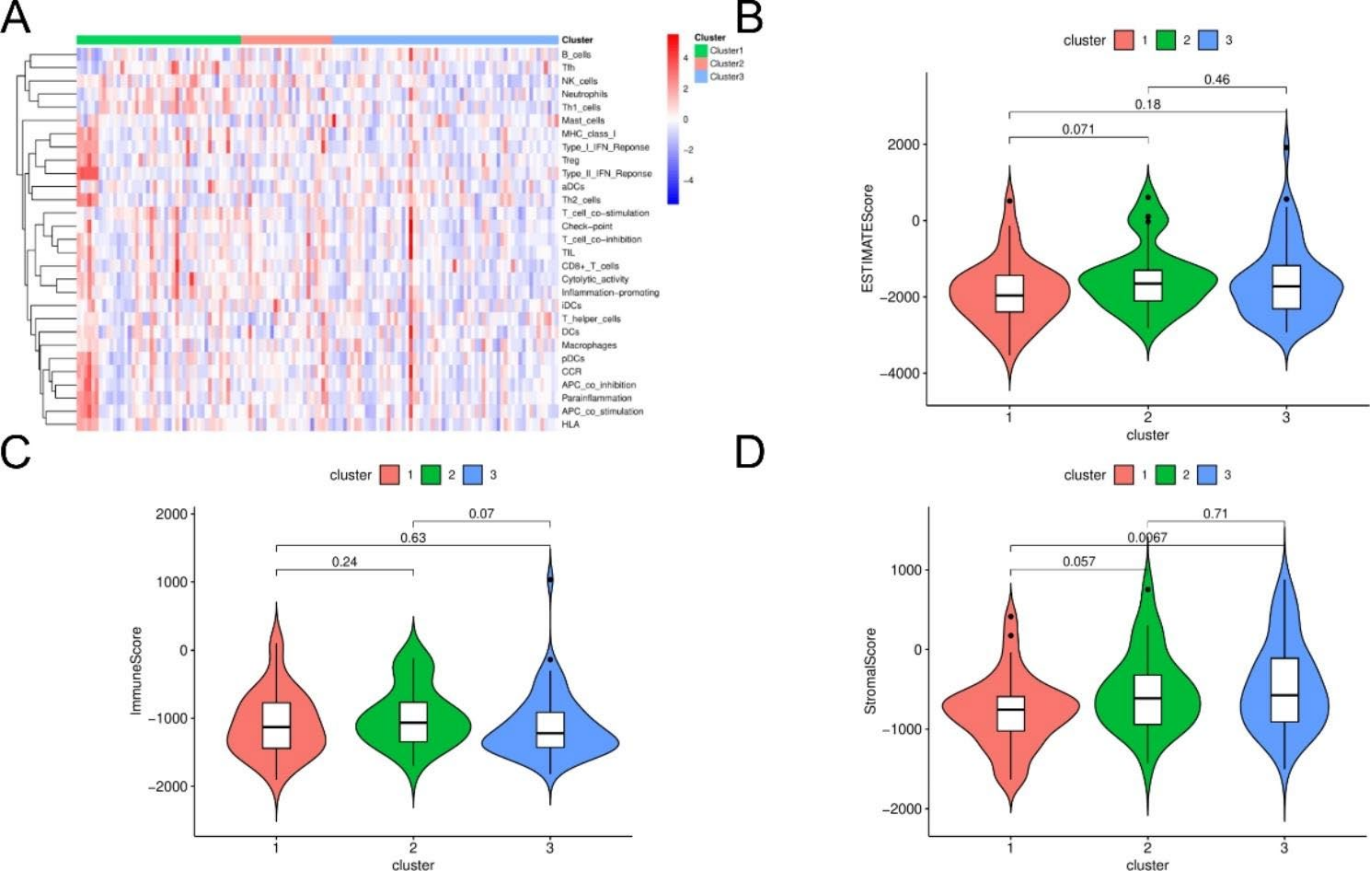
**A**: immune infiltration of different modification patterns; **B-D** different modification patterns ESTIMATE

**Fig. 5:**
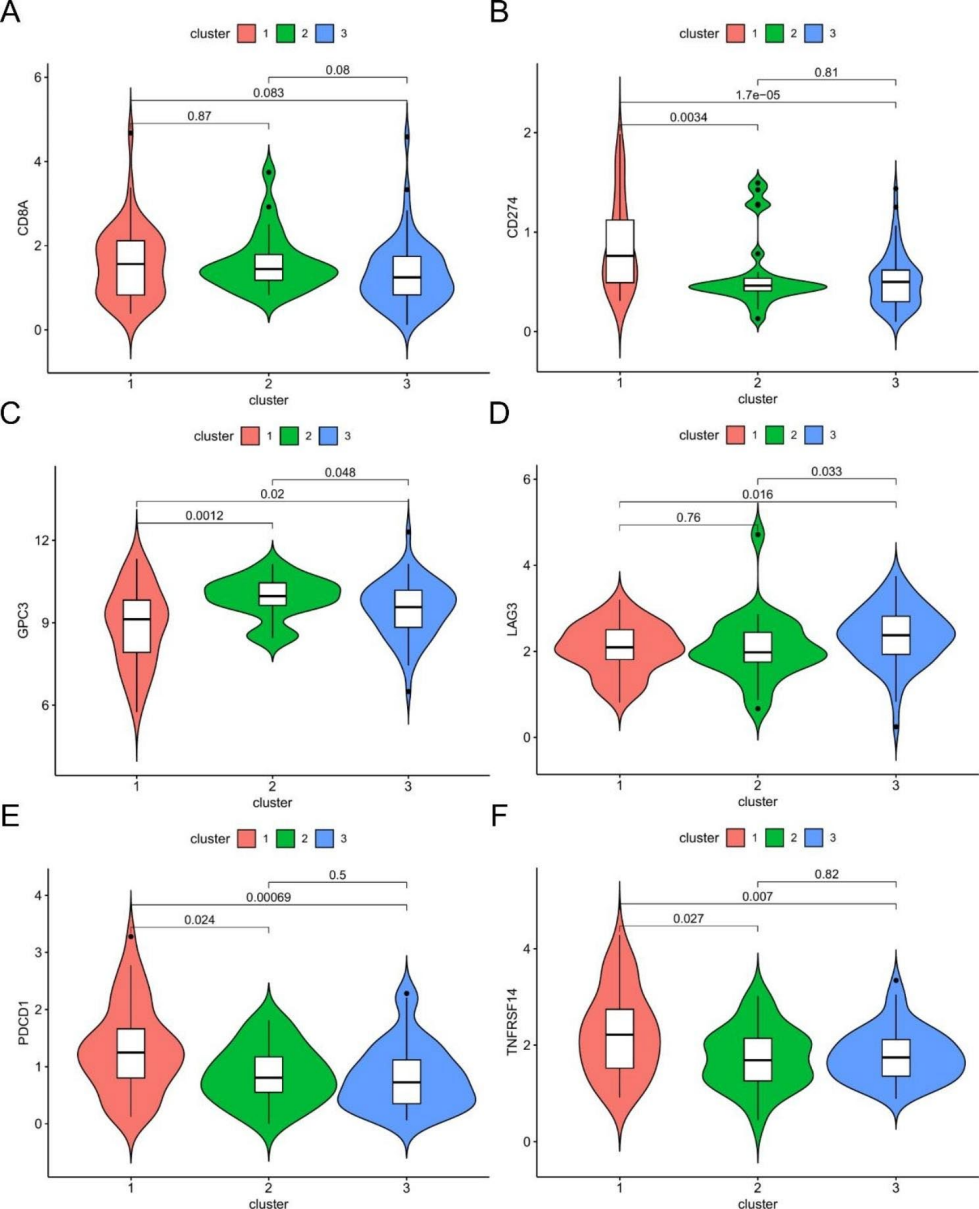
Differential expression of markers such as immune checkpoints with different modification patterns

The infiltration of 29 immune cells was analyzed by ssGSEA in different m6A modification patterns. The results showed that the immune infiltration was significantly different between the different clusters (Fig. 4.A). The results obtained by the ESTIMATE algorithm show a significant difference in StromalScore between cluster3 and cluster1, with Cluster3 having a higher StromalScore. (Fig. 4.B-D). In addition, we analyzed the expression levels of immune checkpoints, immune cell markers and WT metabolites in different clusters [3]. Among the immune checkpoints, cluster1 had higher expression of *CD274* and *PDCD1*, while cluster3 had higher expression of *LAG3*; the star molecule in WT metabolites, *GPC3*, was expressed in the 3 clusters, and cluster2 was higher than the other two groups (Fig. 5).

### Generation of the m6Ascore model

**Fig. 6:**
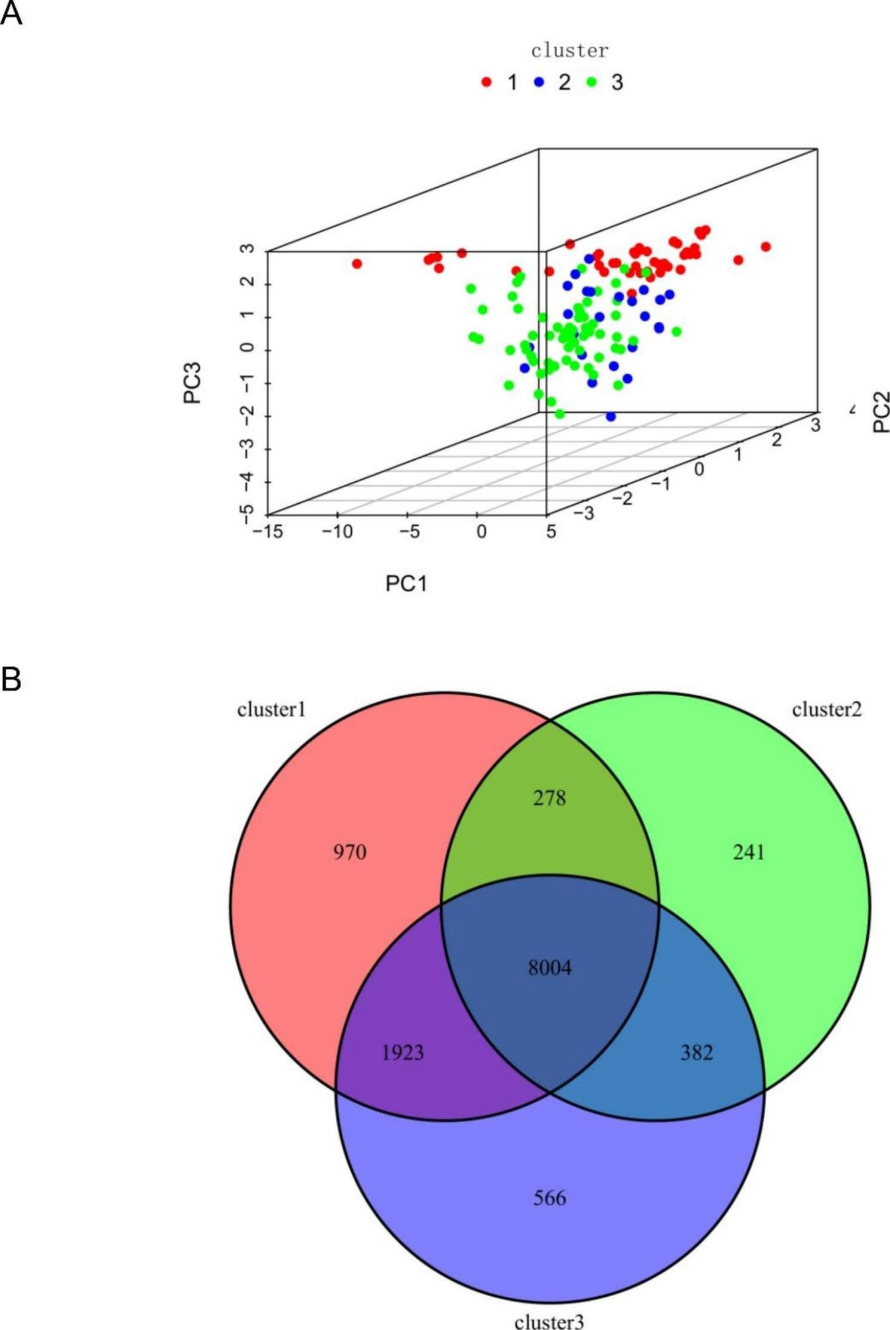
**A**: PCA analysis; **B**: differential expression of tumor and normal tissues under different modification patterns

**Fig. 7:**
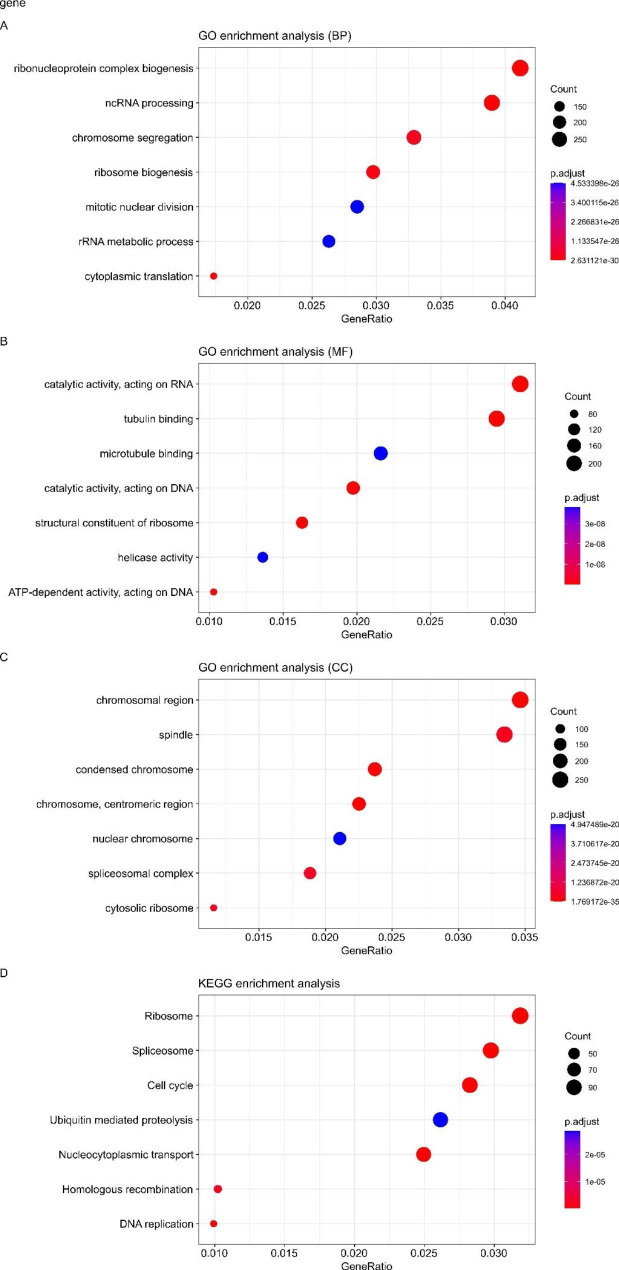
**A-C**: GO enrichment analysis of DEG; **D**: KEGG pathway analysis of DEG

PCA analysis showed that there were different m6A modification patterns in WT patients (Fig. 6). GO enrichment analysis and KEGG pathway analysis of different m6A clusters using the R package “clusterProfiler” showed that DEGs were enriched in biological processes related to tumorigenesis and tumor progression, such as rRNA metabolic processes and cell cycle (Fig. 7.A-D).

**Fig. 8:**
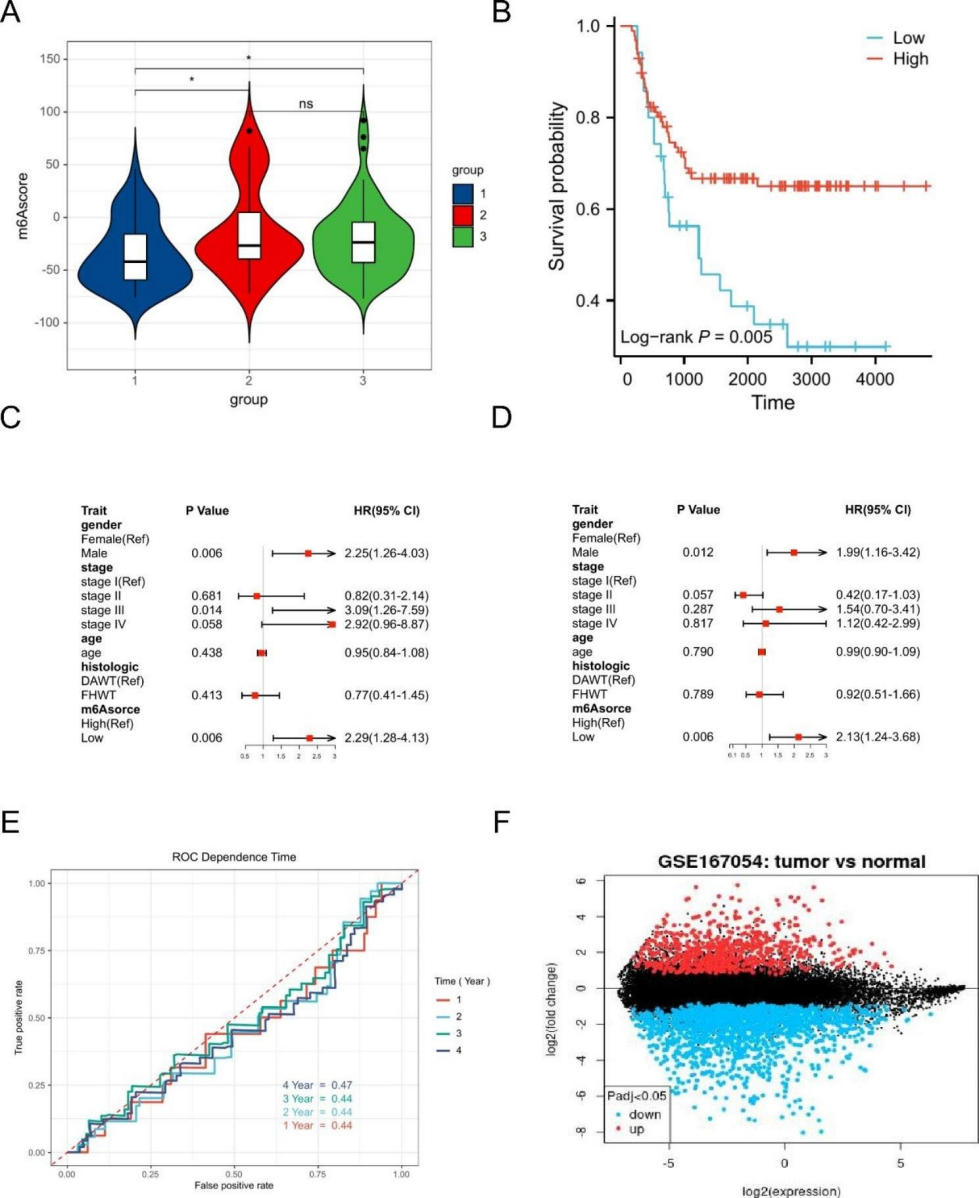
**A**; m6Ascore with different modification patterns; **B**: KM analysis based on m6Ascore; C: univariate independent prognosis; **D**: multivariate independent prognosis; **E**: ROC curve analysis; **F**: Analysis of m6A methylation differences

Univariate Cox regression analysis was used to understand the prognostic value of each DEG, and a total of 284 DEGs with prognostic utility were screened to construct m6Ascore, and the scores were grouped into high and low scores based on the best critical value. The association between m6A modification pattern and m6Ascore was analyzed using analysis of variance, and the results showed that cluster1 had a lower score of m6Ascore (Fig. 8.A). The KM method was used to show that the subgroup with low m6Ascore had a worse prognosis (Fig. 8.B). We used univariate and multivariate Cox regression analysis including gender, age, tumor type, m6Ascore and tumor stage to confirm that m6Ascore was an independent prognostic factor for WT (Fig. 8.C-D). Unfortunately, the ROC curves showed that the predictive power of the m6Ascore model for survival outcomes at 1, 2, 3, and 4 years in the WT patients was poor (Fig. 8.E). We further selected a WT data set of m6A methylation modifications, GSE167054, for differential analysis, and the results showed that m6A modifications differed between normal and WT tissues (Fig. 8.F). This validated the reliability of the m6Ascore model to some extent.

**Fig. 9:**
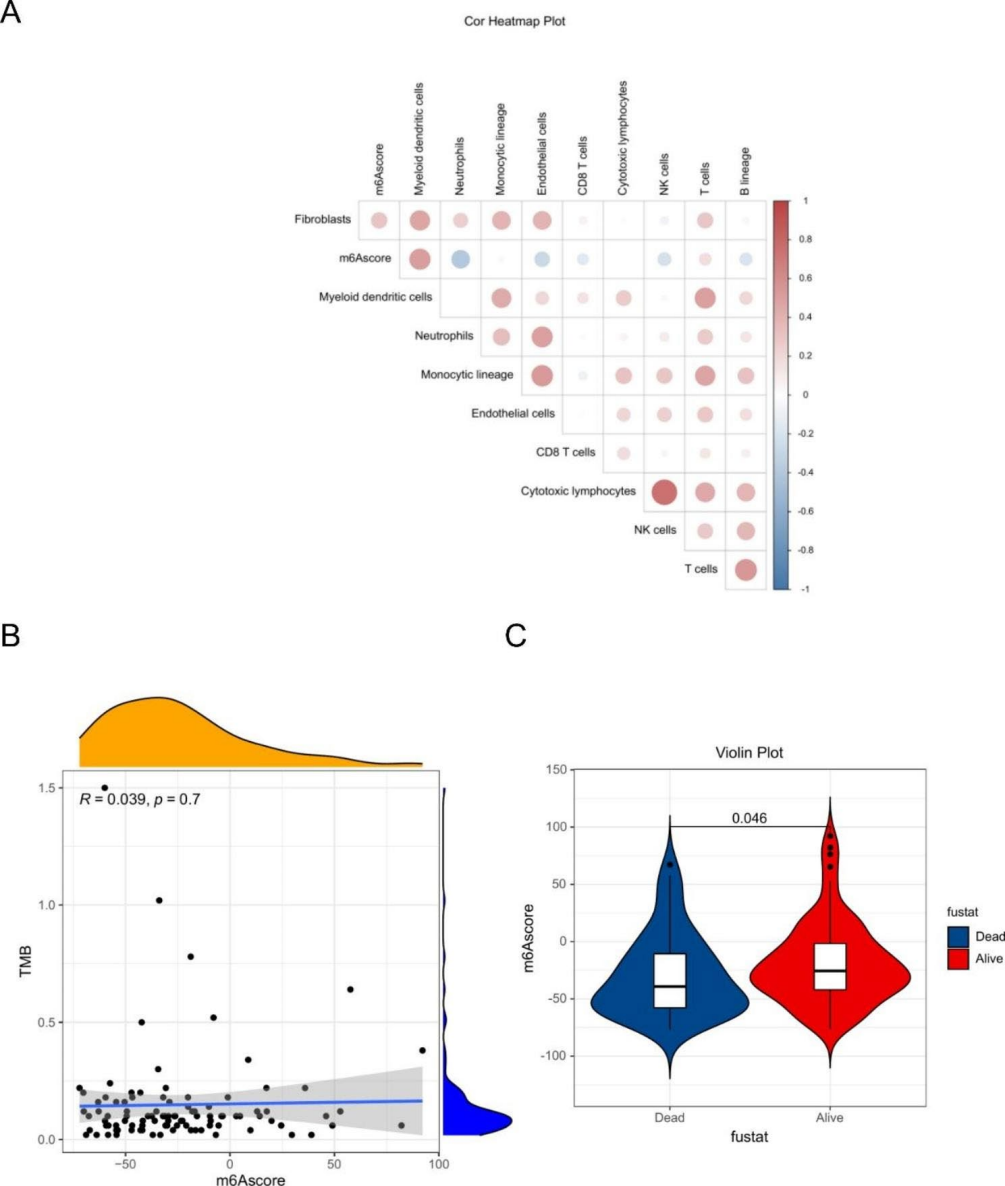
**A**: Correlation between m6Ascore and immune cells; **B**: Correlation between TMB and m6Ascore; **C**: Correlation between m6Ascore and clinical characteristics

**Fig. 10:**
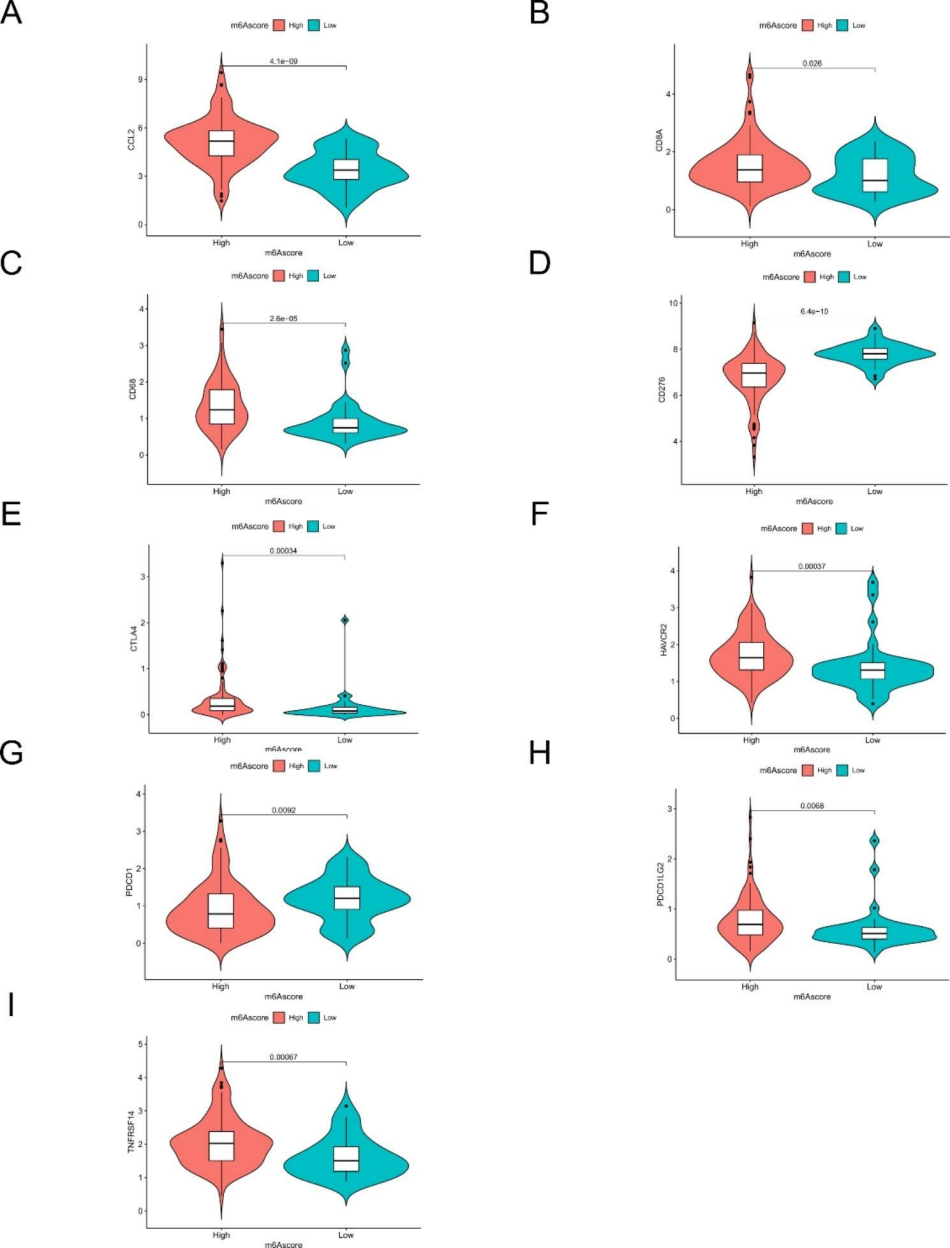
Expression of markers based on m6Ascore grouping

To further understand the potential biological mechanisms of m6Ascore, we analyzed the correlation between m6Ascore and some biological processes. m6Ascore was closely associated with the infiltration of immune cells, such as a significant positive correlation with dendritic cells and T cells, and a significant negative correlation with neutrophils (Fig. 9.A). However, there was no significant correlation between TMB and m6Ascore (Fig. 9.B). In addition, our results showed that patients with low m6Ascore had a higher percentage of death (Fig. 9.C). Our results also showed significant differences in the expression of immune checkpoints, immune cell markers, and tumor metabolic markers in high and low m6Ascore groups, such as *CCL2*, *CD8A*, *CD68*, *CTLA4*, *HAVCR2*, and *PDCD1LG2* were highly expressed in the high m6Ascore group, while two immune checkpoints, *CD276* and *PDCD1*, were highly expressed in the low m6Ascore group (Fig. 10). This suggests that m6Ascore may become a new evaluation index in immune-targeted therapy.

## Discussion

Currently, an increasing number of studies have shown that m6A modifications play an important role in tumorigenesis and progression [31–33]. In general, m6A plays an important role in tumorigenesis and progression by regulating mRNA stability, expression and translation [34]. For example, *METTL3* can accelerate the metastasis and radiation resistance of Glioblastoma by enhancing the stability of *SOX2*. For example, *METTL3* can accelerate the metastasis and radiation resistance of Glioblastoma by enhancing the stability of *SOX2* leading to malignant events [35]; decreased m6A methylation attenuates the expression of the AKT negative regulator *PHLPP2*, while increasing the expression of the AKT positive regulator mTORC2 [36]. The pathogenesis of Wilms Tumor is still not very clear, and recent several multicenter case studies have suggested that m6A modification patterns may contribute to the development and progression of WT [17–19]. However, most of these studies were limited to single or intergenic studies and did not systematically analyze the impact of m6A regulator-related modification patterns on tumor progression and prognosis.

For the first time, we constructed a model based on m6A regulators to quantify the m6A modification patterns of individual patients, further revealing the potential prognostic value and therapeutic guidance of m6A modification and m6A regulators in WT. To investigate the role of m6A modifications in WT, we systematically analyzed the m6A modification patterns of WT samples extracted from public databases. Three different m6A modification patterns in WT were finally identified by an unsupervised clustering approach [37]. The different modification patterns showed significant differences in immune cell infiltration, biological pathways, and prognosis. To further quantify m6A modifications in individual patients, we constructed a model (‘’m6Ascore”) and demonstrated that this model is an independent prognostic factor for WT. Furthermore, our results suggest that m6A modifications are different in different patients and that related modulators may be a novel prognostic marker.

We found that almost all m6A regulators were expressed at significantly higher levels in WT tissues than in normal tissues, and a previous study suggested that the pathogenesis of WT may be related to changes in RNA methylation [9]. This suggests that altered m6A modifications are associated with the pathogenesis of WT. The three different modification patterns obtained by clustering analysis differed in terms of survival outcomes. m6A regulator expression was low but with poor survival gains in cluster2, and m6A regulator expression was higher and with better survival gains in cluster3. These results suggest that the expression levels of m6A regulators are closely related to tumor progression in WT. Considering the heterogeneity among m6A modifications, the m6Ascore model was constructed to quantify the m6A modification patterns of individual WT patients. Our results indicate that m6Ascore is an independent prognostic factor for WT patients. In addition, we found that m6Ascore was strongly associated with immune cell infiltration. m6Ascore was significantly positively correlated with dendritic cells and T cells and significantly negatively correlated with neutrophils.

A large number of existing studies have shown that the tumor microenvironment can not only influence tumor cell growth and metastasis, but also has great importance in therapy [38–40], in particular, the association between TME and methylation modifications and the influence of TME on tumor progression in WT has been reported [41, 42]. We quantified the infiltration of 29 different immune cell types in WT samples by ssGSEA analysis. The results showed a large difference in infiltration between different clusters. Among them, cluster2 had much lower immune infiltration than cluster1 and worse survival. Previous studies have also shown that a high degree of immune infiltration plays an anti-tumor role to some extent [43, 44].

Immune checkpoint inhibitor (ICI) therapies targeting pathways such as PD-1 and PD-L1 have been widely used in the treatment of tumors [45]. In particular, ICI therapies have revolutionized the way cancer is treated, offering new hope to a wide range of patients [46]. Our study found that the expression levels of these molecules were different in different WT patients. A similar situation was also seen in the m6Ascore model.

The present study still has the following limitations. First, due to various limitations, we could only assess immune cell infiltration based on algorithms and lack of actual experimental data. Second, due to the lack of data, there is a lack of corresponding validation cohorts and an inability to directly examine the actual immunotherapeutic response in the high and low m6Ascore groups. Similarly, we do not have enough clinical cohorts to validate the prognostic value of m6Ascore in WT, and prospective studies of large cohorts are lacking.

In this study, we systematically analyzed the expression characteristics of m6A-related regulators in WT. m6A modification patterns play an important role in the mechanism and prognosis of WT. Our study provides practical tools for predicting the prognosis of WT, and this study may help clinical practitioners to identify valid indicators of Wilms Tumor for the poor prognosis of this disease.

### Electronic supplementary material

Below is the link to the electronic supplementary material.

Supplementary Material 1

Supplementary Material 2

Supplementary Material 3

Supplementary Material 4

Supplementary Material 5

Supplementary Material 6

Supplementary Material 7

Supplementary Material 8

## Data Availability

The raw data of this study are derived from the TCGA database (https://portal.gdc.cancer.gov/), the specific sample is TARGET-WT, which are provided on public databases.
